# A traumatic injury mortality prediction (TRIMP) based on a comprehensive assessment of abbreviated injury scale 2005 predot codes

**DOI:** 10.1038/s41598-021-98558-9

**Published:** 2021-11-05

**Authors:** Muding Wang, Guohu Zhang, Degang Cong, Yunji Zeng, Wenhui Fan, Yi Shen

**Affiliations:** 1 Department of Emergency Medicine, Affiliated Hospital of Hangzhou Normal University, Hangzhou, 310015 Zhejiang People’s Republic of China; 2 Department of Emergency Intensive Care Unit, Affiliated Hospital of Hangzhou Normal University, 126 Wenzhou Road, Gongshu District, Hangzhou, 310015 Zhejiang People’s Republic of China; 3 Department of Thoracic Surgery, Affiliated Hospital of Hangzhou Normal University, Hangzhou, 310015 Zhejiang People’s Republic of China; 4 Department of Orthopedic, Affiliated Hospital of Hangzhou Normal University, Hangzhou, 310015 Zhejiang People’s Republic of China; 5 Department of Epidemiology and Health Statistics, School of Public Health, Zhejiang University, Hangzhou, 310058 Zhejiang People’s Republic of China

**Keywords:** Computational biology and bioinformatics, Diseases, Signs and symptoms

## Abstract

Abbreviated Injury Scale (AIS)-based systems such as injury severity score (ISS), exponential injury severity score (EISS), trauma mortality prediction model (TMPM), and injury mortality prediction (IMP), classify anatomical injuries with limited accuracy. The widely accepted alternative, trauma and injury severity score (TRISS), improves the prediction rate by combining an anatomical index of ISS, physiological index (the Revised Trauma Score, RTS), and the age of patients. The study introduced the traumatic injury mortality prediction (TRIMP) with the inclusion of extra clinical information and aimed to compare the ability against the TRISS as predictors of survival. The hypothesis was that TRIMP would outperform TRISS in prediction power by incorporating clinically available data. This was a retrospective cohort study where a total of 1,198,885 injured patients hospitalized between 2012 and 2014 were subset from the National Trauma Data Bank (NTDB) in the United States. A TRIMP model was computed that uses AIS 2005 (AIS_05), physiological reserve and physiological response indicators. The results were analysed by examining the area under the receiver operating characteristic curve (AUC), the Hosmer–Lemeshow (HL) statistic, and the Akaike information criterion. TRIMP gave both significantly better discrimination (AUC_TRIMP_, 0.964; 95% confidence interval (CI), 0.962 to 0.966 and AUC_TRISS_, 0.923; 95% CI, 0.919 to 0.926) and calibration (HL_TRIMP_, 14.0; 95% CI, 7.7 to 18.8 and HL_TRISS_, 411; 95% CI, 332 to 492) than TRISS. Similar results were found in statistical comparisons among different body regions. TRIMP was superior to TRISS in terms of accurate of mortality prediction, TRIMP is a new and feasible scoring method in trauma research and should replace the TRISS.

## Introduction

There are several well-established scores for predicting the outcome of trauma patients. Initially the abbreviated injury scale (AIS)^1^ was introduced in 1971 by the Association for the Advancement of Automotive Medicine, and it has been further developed with major updates in 2005 (AIS_05) and 2008 (AIS_08)^2^. Many AIS based approaches, such as the injury severity score (ISS)^3^, the new ISS (NISS)^4^, and the Exponential injury severity score (EISS)^5^ have been published and suggested as measures of improved prediction accuracy. Particularly the development of injury mortality prediction (IMP)^6^ derived from a combination of respective regressed models for three different variable groups, and the trauma mortality prediction model (TMPM)^7^ greatly enhanced the predictive ability has also shown that the TMPM method outperforms the NISS and the ISS as a predictor of mortality^8^. As IMP and TMPM provide pure anatomical injury score via AIS 1998 (AIS_98) and do not utilize available clinical data.

The dominated AIS_05 of expanded classifications and details has been applied across most countries and regions, and the AIS_98 version is likely to be history. Comparing against AIS_98, the AIS_05 has seen an increase in the number of predot codes by approximately a third around 1300 to more than 1980^9^, and the ISS score has demonstrated more consistency with the actual mortality^9^.

In 1981, the Trauma and Injury Severity Score (TRISS) was created by Champion HR on the basis of anatomical injury (ISS). Physiological reserve, such as age, and physiological responses, such as Glasgow Coma Score (GCS), systolic blood pressure (SBP), and respiratory rate (RR) are introduced to the model, contributing to improved prediction results than ISS^10^. Since its inception, many attempts have been made to update TRISS with the latest version in 2011^11^ through enriched categories from the two to five in terms of age and revised coefficients and variables. However, TRISS inherits the deficiency from ISS only selects patients aged over 14 years. The statistically significant clinical information, such as injury mechanism, mechanical ventilation, and pre-existing diseases is not fully exploited by TRISS.

Considering the AIS_05 predot codes, physiological reserve, and physiological response, this study introduced a model of traumatic injury mortality prediction (TRIMP), that utilizes extra clinical data, and evaluated its results against.

## Methods

### Data source

This was a retrospective cohort study where injured patients with one or more AIS_05 codes hospitalized between 2012 and 2014 were sampled from the National Trauma Data Bank (NTDB) in the United States^12^. Data fields patient demographics, AIS codes and ISS 2005, mechanism of injury (based on ICD-9-CM E-codes), GCS, length of hospital stay, length of Intensive Care Unit (ICU) admission, the total number of days on a mechanical ventilator, in-hospital mortality, and encrypted hospital identifiers. Concerning E-codes, they were mapped from one to six respectively per the following injury mechanisms: stab wound, violence, blunt injury, fall, motor vehicle crash, and firearm wound.

**Figure 1:**
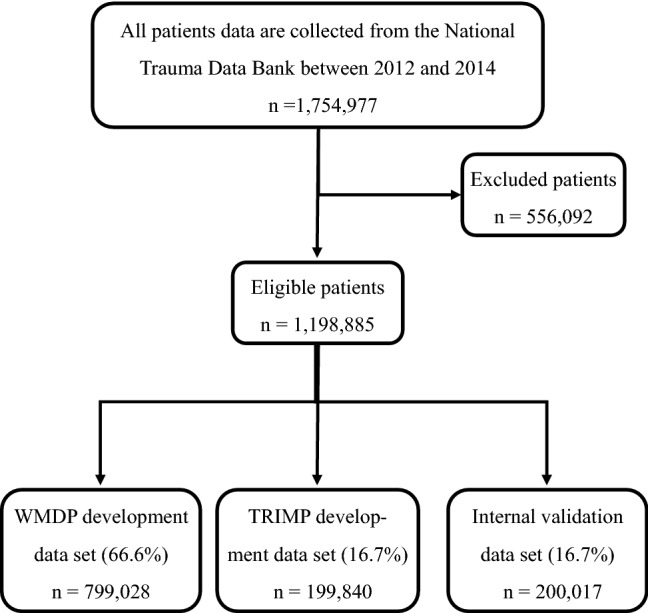
Flowchart for data analyzed. *TRIMP* traumatic injury mortality prediction, *WMDP* weighted median death probability.

The raw included a total of 1,754,977 patients. For each patient an injury description of AIS 2005 is required for both TRIMP and TRISS calculation. Patients with nontraumatic diagnoses (such as drowning, submersion, poisoning, and suffocation), overexertion, or burns (121,257), missing cause of injury (13,083), other missing or invalid data (for fields such as age, gender, length of hospital stay, or outcome) (41,269), age over 89 years (69,478) or below 1 year (35,657), only treatment in the emergency department without being hospitalized (166,990) were excluded from this analysis, as were patients dead on arrival to the hospital (18,581) or transferred to another facility (71,855). Additionally, we also required that patients with either one single injury or multiple injuries have AIS_05 codes other than 9 alone (5282), as otherwise ISS value could not be calculated. At least 500 trauma patients per hospital annually were available (119,393 patients were excluded). The final dataset included 1,198,885 patients admitted to 487 hospitals as shown in Fig. 1.

### TRIMP overview

**Figure 2:**
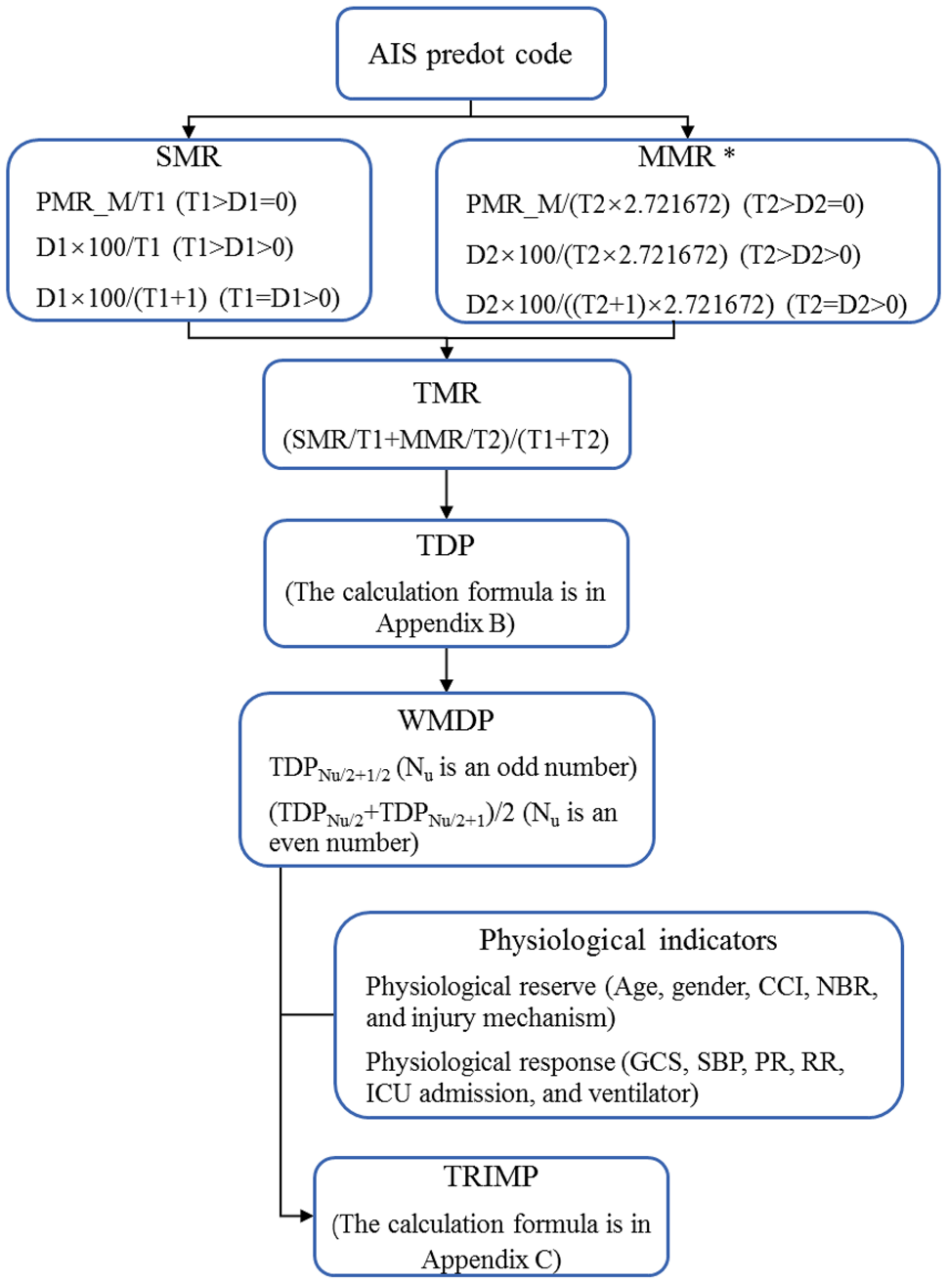
Workflow from AIS to TRIMR. *The average number of injuries per patient was 4.404, 4.404 × 0.618 = 2.721672. $PMR = 0.01202 \times EXP\;(0.0719 \times age)$. D1 and D2 indicate the number of death incidents of a single and multiple injuries with specific AIS predot code, respectively. T1 and T2 indicate the total number of single or multiple trauma cases with specific AIS predot code, respectively. N_u_ is the number of the three worst (maximal) TDP values for specific AIS predot code. *AIS* abbreviated injury scale, *CCI* Charlson Comorbidity Index, *GCS* Glasgow Coma Score, *ICU* intensive care unit, *MMR* multiple trauma mortality rate, *PMR_M* median of possible mortality rate, *SMR* single trauma mortality rate, *TDP* traumatic death probability, *TRIMP* traumatic injury mortality prediction, *WMDP* weighted median death probability.

In this analysis, 66.6% of the dataset was applied to assess the trauma mortality rate (TMR) and weighted median death probability (WMDP) values as per AIS predot codes. A TMR value according to the trend of the crude death rates of each age group in the United States between 2012 and 2014^13^ is adopted, when the true mortality rate of a specific AIS predot code was zero. The TMR and WMDP values were calculated similar to IMP and IMP-ICDX^6,14^, as displayed in Appendices A and B respectively, with their workflow shown in Fig. 2.

16.7% of the dataset was used to evaluate TRIMP. Coefficients of the TRIMP (Table 3) were derived by a probit regression model. The remaining 16.7% of the dataset was not used for the development of WMDP or TRIMP, but for internal validation of the statistical performance of the TRIMP and TRISS models.

### Comorbidity

We used the Charlson Comorbidity Index (CCI) to calculate comorbidities^15^. This is a recognized method to measure the risk of death from post-traumatic comorbid diseases^16^.

### Customized trauma models

This validation dataset enabled to test the performance of the TRISS and TRIMP. TRISS based on the methodology described by Boyd CR^17^. TRIMP was defined in five parts. The first was to incorporate the five most severe (highest) WMDP values as predictors. The second was to determine whether the worst and second-worst traumas were in the same body region (1 for the same, and 0 otherwise). The third was to synthesize the two highest WMDP values into one variable. The fourth introduced physiological reserve indicators, such as injury mechanism, CCI, gender, age, and NBR (as NBR and NBR^0.382^, obtained by fractional polynomial transformation)^18^. The last part added physiological response indicators, such as GCS, vital signs (including SBP, pulse rate, and RR), ICU admission, and mechanical ventilator.

### Statistical analysis

The statistical performance of the trauma models was assessed using the area under the receiver operating characteristic curve (AUC), the Hosmer–Lemeshow (HL) statistics, and the Akaike information criterion (AIC). The AIC serves as a measure of the Kullback–Leibler divergence, which quantifies how closely a statistical model approaches the true distribution. The underlying basis for comparison is that the best model in a particular dataset should be the model with the lowest AIC value. A bootstrapping algorithm of 1000 replications was used to calculate the bias-corrected 95% confidence intervals for the AUC and the HL, where a *p*-value < 0.05 was considered statistically significant. Statistical tool STATA/MP version 14.0 for Windows was used for all analyses. The article was approved from oversight of the Institutional Review Board of Hangzhou Normal University, People’s Republic of China.

### Ethics approval and consent to participate

This study was a retrospective analysis and the data were from the American College of Surgeons’ NTDB dataset. Actually, none of the patients were contacted. It was approved from the examination of the Institutional Review Board of Hangzhou Normal University, People’s Republic of China.

## Results

A total of 1984 AIS predot injury codes from 1,198,885 patients with 4,248,108 injured body regions were studied. Among the dataset, there were 335,470 (28.0%) patients with only one single injury, and the maximum of injured body regions for one patient was 40. The average of injured body regions per patient was 3.47.

We found that the number of injuries per AIS predot code was highly negatively-skewed. On the left tail 138 (7.0%) AIS predot codes appeared less than or equal to 10 times, and on the right side 96 (4.8%) AIS predot codes occurred greater than 10,000 times. The most common AIS predot code (AIS 450203.3: “Rib fracture closed, at least three ribs”) occurred up to 99,590 (8.3%) times, and 50% of the injuries occurred less than 228 times.

66.6% of the dataset was used to develop WMDP and consequently, four AIS predot codes were lost (including four patients). Ultimately, we obtained 1980 WMDP values from different AIS predot codes (See Appendix D). These WMDP values ranged from 0.0009 for a minor trauma that poses minimal threat to life (AIS 730204.1: “Digital nerve injury”) to a value of 2.7469 for a critical trauma (AIS 140216.6: “Brainstem penetrating injury prolonged loss of consciousness with no return”). It was evident that WMDP values were of more precisions than the AIS integers from one to six, for mortality prediction. Interestingly, we noticed that “minor” traumas such as AIS 240207.2: “Injury of the bilateral inner ear or middle ear” were often assigned higher WMDP values, whereas some “severe” traumas, for instance AIS 640462.5: “Complete thoracic spinal cord injury syndrome (paraplegia, no sensory function), no fracture or dislocation”), were associated with relatively low WMDP values. As WMDP values reflect the propensity for death rather than severity of the trauma, these observations were considered appropriate.

Patient demographics were summarized in Table 1. In terms of ethnicity and race, the percentages of Whites and Blacks were 70.5% and 13.7% respectively. The most severe injuries occurred in the limbs (35.3%) and head and neck region (34.2%). Two of the most frequent causes of trauma were fall (44.6%) and motor vehicle accidents (32.6%). Males accounted for 62.1% of the population, and the overall mortality rate of the entire dataset was 3.03% on average.

**Table 1:** Patient demographics.

Patient characteristics	No mechanical ventilator n = 1,054,519 (88.0%)	Mechanical ventilator n = 144,366 (12.0%)
Age, years, median (IQR)	48 (26–68)	45 (26–62)
Male, n (%)	638,989 (60.6)	106,020 (73.4)
**Race, n (%)**		
White, not Hispanic	748,765 (71.0)	96,226 (66.6)
Black or African American	141,221 (13.4)	22,968 (15.9)
Hispanic or Latino	91,951 (8.7)	13,650 (9.5)
Asian	17,181 (1.6)	2215 (1.5)
Native American or Alaskan Native	11,703 (1.1)	2248 (1.6)
Other races	43,698 (4.2)	7059 (4.9)
**Mechanism of injury, n (%)**		
Fall	493,509 (46.8)	41,652 (28.9)
Motor vehicle accident	324,298 (30.8)	66,681 (46.2)
Violence^a^	81,654 (7.7)	7717 (5.3)
Blunt	72,116 (6.8)	7472 (5.2)
Stab	45,895 (4.4)	5692 (3.9)
Firearm	37,047 (3.5)	15,152 (10.5)
**Body region of worst injury, n (%)**		
Head and neck	329,535 (31.2)	80,772 (55.9)
Face	61,266 (5.8)	5626 (3.9)
Thorax	171,898 (16.3)	29,531 (20.5)
Abdomen and pelvic cavity	81,383 (7.7)	12,907 (8.9)
Limbs and pelvis	407,618 (38.7)	15,094 (10.5)
External (skin) and others	2819 (0.3)	436 (0.3)
Injury severity score, median (IQR)	8 (4–10)	17 (10–26)
Dead, n (%)	7552 (0.72)	28,803 (19.95)

^a^Violence indicates to strike or against. *IQR* interquartile range.

Table 2 presents the statistics of both models per body and it is apparent that TRIMP exhibited significantly better discrimination, calibration, and AIC statistics compared against the TRISS model, with exception of the calibration in the second BR. The coefficients of each variable in TRIMP are illustrated in Table 3.

**Table 2:** Performance comparison of TRISS and TRIMP models in different body regions.

Model description	BR	N	AUC (95% CI)	HL stat	AIC
TRISS	All	179,361	0.923 (0.919–0.926)	411.25	32,756.7
	1	61,288	0.921 (0.916–0.925)	130.00	18,079.8
	2	9936	0.932 (0.904–0.960)	3.42	401.9
	3	32,364	0.885 (0.874–0.896)	93.24	5650.5
	4	14,258	0.908 (0.893–0.923)	51.52	2406.9
	5	60,997	0.899 (0.885–0.913)	53.13	5300.4
	6	518	0.920 (0.878–0.962)	11.75	122.2
TRIMP	All	200,017	0.964 (0.962–0.966)	14.3.970	26,13,278.39
	1	68,199	0.959 (0.956–0.961)	4.523.79	14,7749.73
	2	11,080	0.965 (0.9442–0.987)	5.64.75	349.951.0
	3	33,574	0.945 (0.9390–0.951)	167.1820	46,546.9
	4	15,773	0.963 (0.9587–0.969)	5.43.62	2,106.715.9
	5	70,816	0.942 (0.934–0.950)	4436.095	4122.5.3
	6	575	0.9721 (0.9576–0.9876)	1.6071	96.57.3

Compared with TRISS model, TRIMP model of most BRs except calibration at the second BR, has better discriminability, calibration and AIC.

*AIC* Akaike information criterion, *AUC* area under the receiver operating characteristic curve, *BR* body region, *HL stat* Hosmer–Lemeshow statistic.

**Table 3:** TRIMP regression coefficients.

Predictor		Coefficients	Robust std. error	*Z*	*P* >|*z*|	95% CI
WMDP_1_	C_1_	1.74286	0.05855	29.77	0.000	1.62811–1.85762
WMDP_2_	C_2_	0.90205	0.14902	6.05	0.000	0.60999–1.19412
WMDP_3_	C_3_	0.48395	0.08800	5.5	0.000	0.31147–0.65644
WMDP_4_	C_4_	0.24008	0.12063	1.99	0.047	0.00365–0.47652
WMDP_5_	C_5_	0.52620	0.11745	4.48	0.000	0.29600–0.75641
WMDP_1_ × WMDP_2_	C_6_	− 0.14533	0.06401	− 2.27	0.023	− 0.27079 to − 0.01987
Same region	C_7_	− 0.16288	0.04142	− 3.93	0.000	− 0.24407 to − 0.08169
NBR	C_8_	0.09595	0.01685	5.69	0.000	0.06292–0.12897
NBR^0.382^	C_9_	− 1.84530	0.17565	− 10.51	0.000	− 2.18958 to − 1.50103
Age	C_10_	0.04249	0.00102	41.62	0.000	0.04049–0.04449
Gender	C_11_	0.13122	0.03725	3.52	0.000	0.05820–0.20423
CCI	C_12_	0.29517	0.02260	13.06	0.000	0.25087–0.33947
Injury mechanism	C_13_	0.15933	0.01981	8.04	0.000	0.12050–0.19815
GCS	C_14_	− 0.11159	0.00440	− 25.36	0.000	− 0.12022 to − 0.10297
ICU admission	C_15_	0.20514	0.05609	3.66	0.000	0.09521–0.31507
Ventilator	C_16_	1.76084	0.05315	33.13	0.000	1.65666–1.86502
SBP	C_17_	0.39901	0.01841	21.67	0.000	0.36293–0.43509
Pulse rate	C_18_	0.27502	0.01752	15.69	0.000	0.24067–0.30936
RR	C_19_	0.09428	0.01319	7.15	0.000	0.06843–0.12013
Constant	C_0_	− 7.87668	0.22814	− 34.53	0.000	− 8.32384 to − 7.42953

Coefficients for TRIMP model were recalculated based on 199,840 patients. WMDP_1_ is the worst injury (max WMDP value), WMDP_2_ the second worst injury, and so on. Same region indicates a binary variable, which is equal to 1 if the 2 worst traumas are in the same region, 0 otherwise. WMDP_1_ × WMDP_2_ represents the product of the WMDP values for the 2 worst injuries. The code value of gender is set as 1 for male and 0 for female. The code value setting for other variables, see Appendix A. NBR is the number of body regions and CCI is Charlson Comorbidity Index for each injured patient.

*CCI* Charlson Comorbidity Index, *CI* confidence interval, *GCS* Glasgow Coma Score, *ICU* intensive care unit, *NBR* number of body regions, *RR* respiratory rate, *SBP* systolic blood pressure, *WMDP* weighted median death probability.

**Figure 3:**
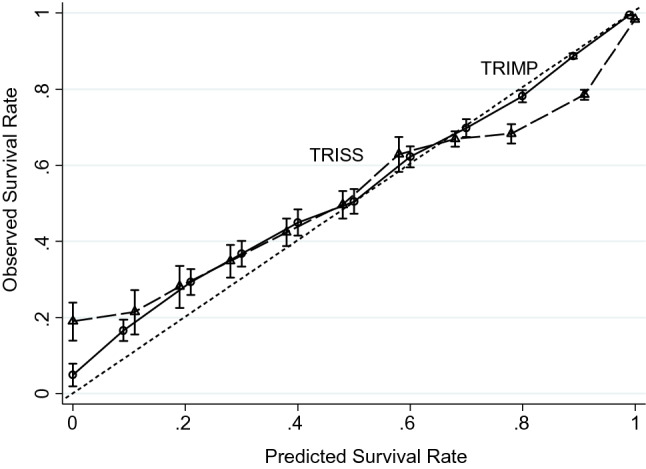
2 Calibration curves for TRIMP and TRISS. The dotted reference lines represent perfect calibration. The 95% binomial confidence intervals for both models are based on the same validation dataset of 200,017 patients. The comparisons of the survival rate of each corresponding calibration point shows that the first calibration point and the last 3 calibration points are statistically significant (*p* < 0.05).

**Figure 4:**
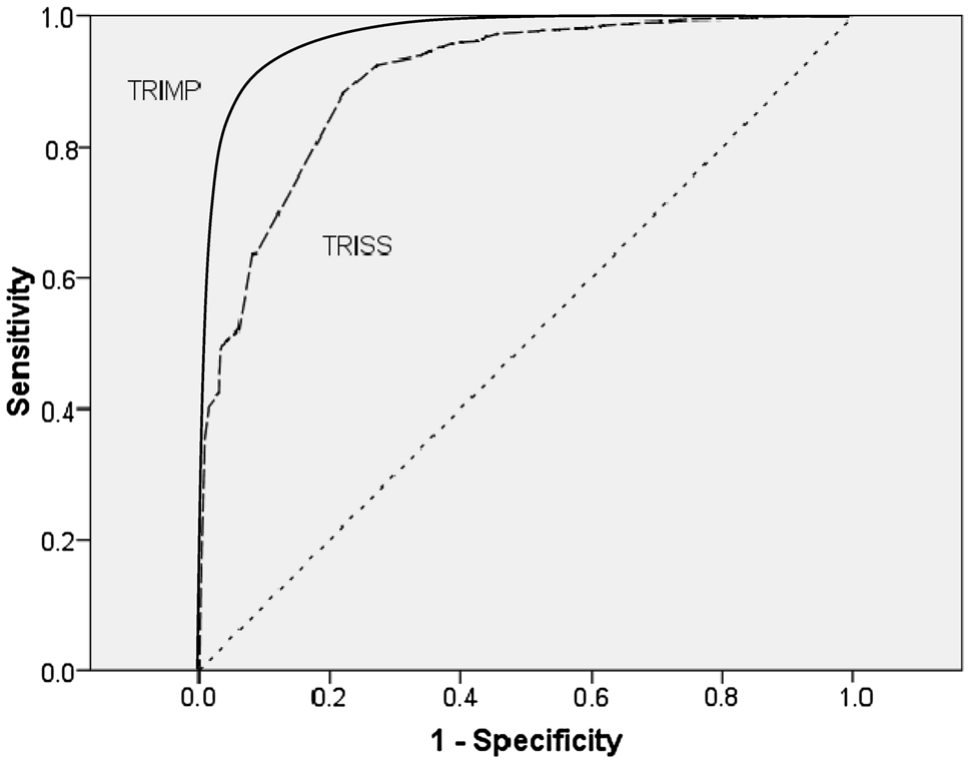
AUC curves for TRIMP and TRISS. A straight line at a 45-degree angle represents standard reference line for the AUC curve.

Figure 3 emphasizes the superiority of TRIMP over TRISS, as the TRIMP survival rates were evenly distributed and close to the dotted reference line. On the other hand, the TRISS survival rates distribution intersected with the dotted reference line. Figure 4 shows that TRIMP provides superior improvement in discrimination compared with TRISS.

## Discussion

With the benefits of hardware and software advancements, we have the ability to work with large datasets. Emerging studies have proved that medical data can be studied by various elaborate computing methods. With improved trauma scoring methods, certain software systems can help to compute and evaluate the severity of disease, from qualitative diagnosis to quantitative diagnosis. As medical costs continue to rise, there is an urgent need for trauma prediction accuracy for both patients and trauma surgeons. It is also of growing interest to stakeholders outside the medical industry. Therefore, we aim to improve the prediction accuracy by digitalization and to reach a stronger quantitative diagnosis, based on existing research such as TMPM, IMP, and IMP-ICDX^6,7,14^.

Since the inception of ISS by Baker and his colleagues in 1974^3^, injury severity evaluation built on multiple BRs has been continuously recognized by medical practitioners all over the world. Obtained from a sum of squares of the three highest AIS values among the six injured body regions could still serve as a fundamental of TRISS^10,11,17^ in spite of its limitations. Following TRISS, we found that TRIMP was far superior in terms of indicators (Table 2, Figs. 3 and 4), for example, 1980 individual WMDP values (differ from one another) exhibited significantly more accuracy and precision than AIS values with variations of only six integer. Specifically, the WMDP values were drawn from research, and the AIS values, nevertheless, were decided by trauma specialists. For small groups of data, AIS values may have advantages to some extent, but it comes to a big dataset, such as information stored in NTDB, empirical research should be recommended for prediction accuracy^19^.

Former research has shown that the IMP derived from the AIS_98 predot code based regression model is superior to the traditional ISS in predicting trauma results^6^. The IMP and traditional ISS models focused on anatomical injuries and disregarded available clinical information such as physiological reserve or physiological response. TRISS was developed further on the basis of ISS by introducing this information, such as age for physiological reserve and GCS, SBP, and RR for physiological response and gave higher accuracy than ISS^10,11^. Still, TRISS could be improved by including more clinical information, and in this study, TRIMP is only compared against TRISS, not IMP or ISS.

Only the mortality probability value of the most severe injury is used in TRIMP, and the coefficient of the most severe injury is approximately 3 times the coefficient of minor injury (results not shown). The interaction of the two most severe WMDPs can cut down the difference in trauma coefficients (Table 3). Usually, trauma surgeons estimate the clinical condition of a patient via one or two of the most severe injuries. Furthermore, TMPM and IMP are based on the notion that the five most severe injuries of a patient largely determine the probability death^6,7^. In this dataset, only five coefficients of the most severe injury per patient were statistically significant (Table 3).

Extra clinical indicators as variables can often improve the prediction accuracy as the development of TMPM, IMP, and TRISS all suggested^6,7,10,11,17^. This study indicated that when GCS, SBP, RR, age and admission of ICU are considered as variables, TRIMP significantly outperforms TRISS. Accordingly, TRIMP is calculated as the sum of the five highest WMDP values and included more variables for physiological reserve, e.g. age, gender, CCI, NBR, and injury mechanism and physiological response, such as GCS, ICU admission, mechanical ventilation, and vital signs (Table 3). The prediction results of TRIMP were satisfactory (Table 2, Figs. 3 and 4) especially when gender, CCI, and age for the physiological reserve. The CCI has been **r**egarded as an independent variable for mortality prediction^16^, the mechanism of injury and NBR can be considered as the indirect indicators of physiological reserve. The addition of injured NBR to the model helps predict traumatic death (or survival)^6,14^. In comparison parametric regression, non-parametric regression, where age and GCS were not classified, illustrated the relation of age and GCS to the traumatic mortality^16,20^. Supplementary variables, such as ICU admission, mechanical ventilation, were contributory factors to forecasting trauma outcomes^14^.

There are several indications for ICU admission of injury patients, for instance, life support after cardiopulmonary resuscitation, mechanical ventilation, and post-trauma monitoring and treatment. Particularly in terms of mechanical ventilation, there are indications, for example, unconsciousness, and loss of spontaneous breathing. Generally, patients who require mechanical ventilation and/or admission to the ICU are severely injured. These indications could be utilized as an indirect physiological response to trauma, as existing findings have confirmed^14^.

This study applied all available data to evaluate TRIMP, unlike other studies that evaluate blunt and penetrating injuries independently^10,11,17^. When their results are calculated separately, predictive performance of penetrating injuries is better than that of blunt injuries^10,11,17^. If a separate evaluation is required, the evaluation can be conducted by the equations derived from this research. The AUCs of blunt injury and penetrating injury are 0.961 and 0.978, respectively—details are not presented in this paper. Injury mechanism coding can be used to correct their results; thus, it is not necessary to evaluate with two separate equations.

The AIS_98 based TMPM and IMP are now outdated trauma score methods due to the popularity of the AIS_05. AIS_05 predot codes provide several classifications third more than AIS_98 predot codes. Theoretically, AIS_05 based TRIMP gave more precision and accuracy in predicting mortality by fully exploiting useful clinical information. The absolute AUC value of TRIMP based on AIS_05 was much more significant than that of IMP based on the AIS_98 when different AIS versions are compared. We evaluated each AIS_05 based WMDP value via statistical and mathematical approaches similar to IMP and IMP-ICDX^6,14^. On the basis of anatomic injury, physiological reserve, and physiological response were taken into account in TRIMP, and this unique approach presented in this study could prediction power by a much intuitive quantitative diagnosis and is easier for the clinicians to accept. AIS_05 based WMDP values were calculated for predicting trauma probability, these values might change but could be recalculated as in line with the updates of AIS versions.

Theoretically, when the death (survival) probability (WMDP value) of each trauma is obtained, it will be possible for the clinicians to assess the trauma severity reliably. In other words, after the correct diagnosis of an individual patient is loaded as electronic medical records, the corresponding probability of death (survival) can be automatically calculated by a programmed script. This could be preliminary research to be conducted by artificial intelligence to benefit clinicians. This calculation method can be extended for all clinical diagnoses, e.g., different ICD-10-CM codes for evaluation of death or survival probability for individual patient.

## Conclusions

TRIMP was superior to TRISS in better discrimination, calibration, and AIC and gave a more accurate prediction of mortality. In summary, TRIMP is a new and feasible scoring method in trauma research and should replace the TRISS.

## Supplementary Information

Supplementary Information 1.

Supplementary Information 2.

Supplementary Information 3.

Supplementary Information 4.

## Data Availability

The data that support the findings of this study are available from NTDB databases of American College of Surgeons, which is publicly available.

## Abbreviations

AIC: Akaike information criterion
AIS: Abbreviated injury scale
AUC: Area under the receiver operating characteristic curve
BR: Body region
CCI: Charlson Comorbidity Index
CI: Confidence interval
GCS: Glasgow Coma Score
HL: Hosmer–Lemeshow
ICD-10-CM: International Classification of Diseases, Tenth Revision, Clinical Modification
ICU: Intensive care unit
IMP: Injury mortality prediction
IMP-ICDX: Injury mortality prediction for ICD-10-CM
IQR: Interquartile range
ISS: Injury severity score
MMR: Multiple trauma mortality rate
MVC: Motor vehicle crash
NBR: Number of body region
NISS: New injury severity score
NTDB: National Trauma Data Bank
PMR: Possible mortality rate
PR: Pulse rate
Ps: Survival probability
RR: Respiration rate
SBP: Systolic blood pressure
SD: Standard deviation
SMR: Single trauma mortality rate
TDP: Trauma death probability
TMPM: Trauma mortality prediction model
TMPM-ICD10: Trauma mortality prediction model for ICD-10-CM
TMR: Traumatic mortality rate
TRIMP: Traumatic injury mortality prediction for AIS 2005
TRISS: Trauma and injury severity score
WMDP: Weighted median death probability
