# Supplementary material for: A traumatic injury mortality prediction (TRIMP) based on a comprehensive assessment of abbreviated injury scale 2005 predot codes
Source: Sci Rep. 2021 Nov 5;11:21757. doi: 10.1038/s41598-021-98558-9 (PMC8571365; doi:10.1038/s41598-021-98558-9)
Supplement: Supplementary file 1 — Supplementary Information 1. [file 41598_2021_98558_MOESM1_ESM.doc]

**Appendix a**

**The** **calculation method of TMR values**

Based on the functional relationship between each age group in the United States and its corresponding population crude mortality from 2012 to 2014 [^12^](#OLE_LINK12), the trend-line function: $y=0.01202\times EXP(0.0719\times age)$, EXP() is an exponential function based on *e* (*e* = 2.718282…), the coefficient of determination (R^2^) is 0.9811. The corresponding possible mortality rate (PMR, *y*) can be calculated according to different ages (from 1 to 89 years old). PMR can be used as a value that the actual mortality rate for different AIS predot codes equals to zero. By way of example, a 48-year-old patient, PMR = 0.01202 × EXP(0.0719 × age) = 0.3790893 (%). The study introduces the golden ratio, 0.618, as a parameter. Because not all injuries lead to death.

In this study, A total of 66.6% of the data were used to calculate trauma mortality rate (TMR). First, for the calculation of a single trauma mortality rate (SMR). In single injury cases, specific AIS predot code deaths (D1) divided by the total number of cases (T1) and then multiplied by 100, which is SMR = D1 / T1 × 100. If D1 = 0, this study sets the mortality rate as the median of PMR (PMR_M) divided by the total number of cases (T1), SMR = PMR_M / T1. If D1 = T1, we assume to added the cases with one survivor, SMR = D1 / (T1+1) × 100.

Second, for the calculation of multiple trauma mortality rate (MMR) (In this study multiple injury patients have an average of 4.404 injuries per patient). In multiple injury cases, specific AIS predot code deaths (D2) divided by the total number of incidents (T2) multiplied by 100 and then divided by 4.404, MMR = D2 / T2 × 100 / (4.404 × 0.618). If D2 = 0, this study sets the mortality rate as the median of PMR (PMR_M) divided by 4.404 and the total number of cases (T2), MMR = PMR_M / (T2 × 4.404 × 0.618). If D2 = T2, we assume to added the cases with one survivor, MMR = D2× 100 / ((T2+1) × 4.404 × 0.618).

Third, merge single and multiple trauma mortality rates of specific AIS predot code as TMR, $TMR=\left(SMR\times T1+MMR\times T2\right)/\left(T1+T2\right)$. Although there is the same TMR for some different AIS predot codes, to ensure that every TMR value is different, we make sure that every TMR is successively decremented by 0.0000001 according to the size of the total number of trauma regions. In the end, a total of 1,980 TMR values for different AIS codes are listed in Appendix [D](../4.%20Appendix%20D.xls).

**Setting the coded value of each variable**

In this paper, for multi-category variables, the corresponding code values are set according to different mortality rates, as shown in Table [A](#Table_A). For each binary classification variable (such as ICU admission and mechanical ventilator), the corresponding code values are set as 1 for presence and 0 for absence.

| **Table A**. The coded value of each variable | | | | | | |
| --- | --- | --- | --- | --- | --- | --- |
| **Coded value** | | **SBP** | **RR** | **PR** | **Injury mechanism** | |
| 1 | 100-174 | | 15-24 | 60-100 | | Stabbing |
| 2 | >174 | | 25-29 | 101-129 | | Violence [*](#OLE_LINK4) |
| 3 | 75-99 | | 10-14 | 40-59 | | Blunt injury |
| 4 | 50-74 | | >29 | >129 | | MVC |
| 5 | 0-49 | | 0-9 | 0-39 | | Fall |
| 6 |  | |  |  | | Gunshot |
| MVC: Motor vehicle crash, PR: Pulse rate, RR: Respiratory rate, SBP: Systolic blood pressure.  * Violence means to strike or against. | | | | | | |
