# Supplementary material for: A traumatic injury mortality prediction (TRIMP) based on a comprehensive assessment of abbreviated injury scale 2005 predot codes
Source: Sci Rep. 2021 Nov 5;11:21757. doi: 10.1038/s41598-021-98558-9 (PMC8571365; doi:10.1038/s41598-021-98558-9)
Supplement: Supplementary file 2 — Supplementary Information 2. [file 41598_2021_98558_MOESM2_ESM.doc]

**Appendix B**

**Calculating WMDP values**

TMR value replaces each injury AIS predot code. This text mainly adopts 1,980 TMR values of possible injury AIS predot codes, the corresponding BR codes and GCS as fundamental predictors to created three separate probit regression models, respectively. Comprehensively 6 additional variables were employed in three models to decrease the variance: number of body region (NBR), age (as age^2^ and age^2^ × ln(age), suggested by fractional polynomial analysis [^17^](#OLE_LINK17)), gender, mechanical ventilator, injury mechanism and independent influence of hospital. (*P*_TMR_ = *P*(death)1, *P*_GCS_ = *P*(death)2, and *P*_BR_ = *P*(death)3). The mathematical expressions were as follows:

$$\begin{matrix} P(death)1=\Phi\left(\alpha_{0}+\alpha_{1}\sum_{r=1}^{1980} TMR_{r}+\alpha_{2}\sum_{i=1}^{40} NBR_{i}+\alpha_{3}gender+\alpha_{4}\right.age^{2} \\ \left.+\alpha_{5}(age^{2})\ln(age)+\alpha_{6}ventilator+\alpha_{7}\sum_{j=1}^{6} mechanism_{j}+\alpha_{8}\sum_{k=1}^{487} H_{k}\right) \end{matrix}$$

$$\begin{matrix} P(death)2=\Phi\left(\beta_{0}+\beta_{1}\sum_{s=3}^{15} GCS_{s}+\beta_{2}\sum_{i=1}^{40} NBR_{i}+\beta_{3}gender+\beta_{4}age^{2}\right. \\ \left.+\beta_{5}(age^{2})\ln(age)+\beta_{6}ventilator+\beta_{7}\sum_{j=1}^{6} mechanism_{j}+\beta_{8}\sum_{k=1}^{487} H_{k}\right) \end{matrix}$$

$$\begin{matrix} P(death)3=\Phi\left(\gamma_{0}+\gamma_{1}\sum_{t=1}^{9} BR_{t}+\gamma_{2}\sum_{i=1}^{40} NBR_{i}+\gamma_{3}gender\right.+\gamma_{4}age^{2} \\ \left.+\gamma_{5}(age^{2})\ln(age)+\gamma_{6}ventilator+\gamma_{7}\sum_{j=1}^{6} mechanism_{j}+\gamma_{8}\sum_{k=1}^{487} H_{k}\right) \end{matrix}$$

TMR*_r_*, *r* = 1, ..., 1,980 is a binary indicator variable for each of the 1,980 modified AIS predot codes.

GCS*_s_*, *s* = 3, ..., 15 is a binary indicator variable for each of the 13 GCS values.

BR*_t_*, *t* = 1, ..., 9 represents a binary indicator variable for each of the 9 BR codes.

NBR*_i_*, *i* = 1, ..., 40 is a binary indicator variable for each of the 40 NBR values.

mechanism*_j_*, *j* = 1, ..., 6 represents a binary indicator variable for the mechanism of injury.

H*_k_*, *k* =1, ..., 487 is a binary indicator variable for each of the 487 hospitals and Φ is the cumulative distribution function.

We then combine five variables: *P*_TMR_, *P*_GCS_, *P*_BR_, AIS, and TMR, to estimate each traumatic death probability (TDP) value:

$$\begin{matrix} TDP=\ln\left(\ln\left(\frac{P_{TMR}+3.70}{TMR}+1\right)\times\right.\left(\ln\left(\frac{P_{GCS}+3.85}{TMR}+1\right)/\ln\left(\frac{P_{BR}+3.81}{TMR}+1\right)\right)^{1.618} \\ \left.\times TMR\times\ln\left(\frac{AIS^{3}}{2}+1\right)^{0.309}\times\ln\left(\frac{TRN^{3}}{2}+1\right)^{0.191}+1\right) \end{matrix}$$

where TMR round number (TRN) = TMR^0.5^ + 0.5 (Round numbers). The TMR values are collapsed into 6 integer values according to mathematic characteristics, whose values are similar to the AIS severity codes. TRN is set to 6 if it is greater than 6. In order to ensure that each variable is not negative, the corresponding constants (3.70, 3.85, and 3.81) are added to *P*_TMR_, *P*_GCS_, and *P*_BR_, respectively.

This research estimates the weighted median of the three worst (maximal) TDP values for each trauma AIS predot code, namely, which is called Weighted Median Death Probability (WMDP). The formula is as follow:

$WMDP_{u}=TDP_{\frac{N_{u}+1}{2}}$ , when N_u_ is an odd number.

$WMDP_{u}=\frac{TDP_{N_{u}/2}+TDP_{N_{u}/2+1}}{2}$ , when N_u_ is an even number.

where N_u_ is the number of the three worst (maximal) TDP values or whatever are lesser than the third worst TDP value for specific AIS predot code.

In this study, A total of 66.6% data was applied to develop 1,980 WMDP values (Appendix [D](../4.%20Appendix%20D.xls)), the median value of 1,296 TDPs (65.4%, and 79.43% of the BR involved) in the AIS predot codes was greater than the mean. The median of 588 TDPs (29.7% and the involved BR accounting for 20.56%) was lesser than the mean and the remaining of 96 TDPs was equal to the mean. To sum up, most of the data in this research were non-normal distribution, so that the median of TDP was taken as the final value (i.e., WMDP).
