# Supplementary material for: A traumatic injury mortality prediction (TRIMP) based on a comprehensive assessment of abbreviated injury scale 2005 predot codes
Source: Sci Rep. 2021 Nov 5;11:21757. doi: 10.1038/s41598-021-98558-9 (PMC8571365; doi:10.1038/s41598-021-98558-9)
Supplement: Supplementary file 3 — Supplementary Information 3. [file 41598_2021_98558_MOESM3_ESM.doc]

**Appendix C**

**Estimating TRIMP**

It is necessary to replace AIS codes with their respective WMDP values (Appendix [D](../4.%20Appendix%20D.xls)). The WMDP values are sorted by the severity among patients who sustained multiple injuries. We apply 16.7% of the data (not used as a development of WMDP) to estimate the coefficient values of TRIMP with the logistic regression method. In order to assess TRIMP probability of death for an individual patient, the following formula is deduced: $\begin{matrix} P\left(death\right)=\Lambda\left(C_{0}+\sum_{i=1}^{5} C_{i}I_{i}+C_{6}I_{1}I_{2}+C_{7}S+C_{8}NBR\right.+C_{9}NBR^{0.382}+C_{10}age \\ +C_{11}gender+C_{12}CCI+C_{13}\sum_{j=1}^{6} mechanism_{j}+C_{14}\sum_{k=3}^{15} GCS_{k}+C_{15}ICU \\ +C_{16}ventilator+C_{17}\sum_{l=1}^{5} SBP_{l}\left.+C_{18}\sum_{m=1}^{5} PR_{m}+C_{19}\sum_{n=1}^{5} RR_{n}\right) \end{matrix}$where *P*(death) is the mortality predicted by TRIMP and Λ is the cumulative distribution function of the logistic distribution, and *I*_1_, ..., *I*_5_ are WMDP values for the 5 worst injuries, ordered with the highest WMDP value (worst injury) first, the second highest WMDP value second, up to the fifth worst injury. *I*_1_*I*_2_ represents the interaction of WMDP values for the worst two injuries. *S* is an indicator variable equal to 1 if the two worst injuries are in the same body region, 0 otherwise. NBR is the number of body regions and CCI is Charlson Comorbidity Index in each injured patient. The code value of gender is set as 1 for male and 0 for female.

The code value setting for other variables, see Appendix [A](../4.%20Appendix%20A.doc). *C*_0_, ..., *C*_19_ are coefficients in Table [3](../3.%20Table%203.doc).

In order to reflect the actual effect of survival probability (Ps), the probability of death for each patient is converted into an actual survival rate, similar to the calculation method of TRISS [^17^](#OLE_LINK17). The calculation formula is below:

where EXP() is an exponential function based on *e* (*e =* 2.718282···).

In fact, it is very easy and simple to calculate the death probability of TRIMP. We can calculate TRIMP value for any trauma data containing AIS predot code. For individual patients, the calculation program set by Excel in Appendix [D](../4.%20Appendix%20D.xls) can be used. While for the evaluation of large data, we use Visual FoxPro 6.0 language to create a program for calculating TRIMP values, which can be provided by the corresponding author if needed.

If readers calculate TRIMP values by their own programming, their trauma databases must complete the following steps before evaluation: First, adding WMDP values in Appendix [D](../4.%20Appendix%20D.xls) of this paper to the respective AIS predot code; Second, ranking the injury severity of each patient according to the size of WMDP values. Third, determining if the two most severe traumas are in the same region (if it is, it is expressed as 1, 0 otherwise); Fourth, calculating NBR and CCI on each patient; Fifth, other variables are set according to Table A in Appendix [A](../../IMP_2005_3Y/4.%20Appendix%20A.doc); Sixth, referring to the corresponding coefficients of each variable in Table [3](../3.%20Table%203.doc) (set another database). Finally, TRIMP value was calculated and converted into survival probability according to the above formula.
